# Incentives for non-physician health professionals to work in the rural and remote areas of Mozambique—a discrete choice experiment for eliciting job preferences

**DOI:** 10.1186/s12960-015-0015-5

**Published:** 2015-04-26

**Authors:** Ayako Honda, Ferruccio Vio

**Affiliations:** Health Economics Unit, School of Public Health and Family Medicine, University of Cape Town, Cape Town, South Africa; Maputo, Mozambique

**Keywords:** Discrete choice experiment, Job preferences, Health worker motivation, Retention, Non-physician health professionals, Mozambique

## Abstract

**Background:**

Successfully motivating and retaining health workers is critical for the effective performance of health systems. In Mozambique, a shortage of health care professionals and low levels of staff motivation in rural and remote areas pose challenges to the provision of equitable health care delivery. This study provides quantitative information on the job preferences of non-physician health professionals in Mozambique, examining how different aspects of jobs are valued and how health professionals might respond to policy options that would post them to district hospitals in rural areas.

**Methods:**

The study used a discrete choice experiment (DCE) to elicit the job preferences of non-physician health professionals. Data collection took place in four Mozambique provinces: Maputo City, Maputo Province, Sofala and Nampula. DCE questionnaires were administered to 334 non-physician health professionals with specialized or university training (‘mid-level specialists’ and N1 and N2 categories). In addition, questionnaires were administered to 123 N1 and N2 students to enable comparison of the results for those with work experience with those without and determine how new N1 and N2 graduates can be attracted to rural posts.

**Results:**

The results indicate that the provision of basic government housing has the greatest impact on the probability of choosing a job at a public health facility, followed by the provision of formal education opportunities and the availability of equipment and medicine at a health facility. The sub-group analysis suggests that job preferences vary according to stage of life and that incentive packages should vary accordingly. Recruitment strategies to encourage non-clinical professionals to work in rural/remote areas should also consider birthplace, as those born in rural/remote areas are more willing to work remotely.

**Conclusion:**

The study was undertaken within an overarching project that aimed to develop incentive packages for non-physician health professionals assigned to work in remote/rural areas. Based on the DCE results, the project team, together with the Mozambique Ministry of Health, has developed a range of health workforce retention strategies focusing on the provision of housing benefits and professional development opportunities to be utilized when assigning non-physician health professionals to rural/remote areas.

## Background

Successfully motivating and retaining health workers is critical for the effective performance of health systems. In most countries, fewer health professionals work in rural and remote areas than in urban and city areas, and in low- and middle-income countries (LMICs), the inequitable distribution of health professionals is even more pronounced due to a lower absolute number of health professionals [1]. The mal-distribution of health professionals makes it difficult for people in underserved areas to access health services and consequently maintain good health. The lack of health professionals adversely affects the quality and quantity of the health care services available and undermines progress towards universal health coverage (UHC), which aims to ensure equitable access to quality health services for all [2,3].

Education, regulation, financial incentives and professional and personal support have been identified as being particularly important factors in the attraction and retention of health professionals to rural and underserved areas [1]. To date, a variety of strategies—quite often packages of the different factors—have been used to respond to the complex nature of the problem [2,4]. However, more knowledge is required on the following: how different types of retention and motivation strategies work in LMIC settings, the effective mixing of strategies in specific contexts and the contextual factors most influencing intervention success [1,2,5].

In Mozambique, a shortage of health care professionals and low levels of staff motivation in rural and remote areas pose challenges to the provision of equitable health care delivery [6,7]. While the public sector health workforce has grown steadily in Mozambique from 15 339 workers in 2000 to 35 790 workers in 2011, the number of health workers per head of population remains one of the lowest in Sub-Saharan Africa (there were 64.5 doctors, nurses and midwives per 100 000 population in 2011), particularly due to a shortage of mid-range health workers, including non-physician health professionals such as nurses and midwives [8]. The shortage is further compounded by a mal-distribution of health professionals between urban and rural areas, which exacerbates equitable access to quality health care services for the rural population. In Mozambique, despite a lack of basic infrastructure in rural and underserved areas (e.g. drinking water, sanitation facilities, electricity) and limited road access during the rainy season, comprehensive strategies to encourage public sector health professionals to accept deployment to rural, underserved areas are yet to be developed [8]. Consequently, it is crucial for the government to develop strategies to motivate and retain health care professionals, particularly in rural and remote areas, where more than 70% of the population live.

The study aims to provide quantitative information on the job preferences of non-physician health professionals in Mozambique, examining how different aspects of jobs are valued and how health professionals might respond to policy options that would post them to district hospitals in rural areas. At the commencement of the study, the Medical Association of Mozambique (Associação Médica de Moçambique) had begun negotiating a package of salary improvements and benefits for relocated workers with the government of Mozambique. Because these negotiations affected work conditions for medical doctors and medical students, we chose to focus our study on the under-represented interests of non-physician health workers. University-graduate-level non-physician health professionals are currently experiencing the most rapid increase in numbers, partly due to an increase in the middle class population, who can afford higher education, and partly due to a strong motivation for higher education among public sector health professionals, for whom education is linked to career advancement [8]. In addition, the number of universities, both public and private, providing higher education opportunities for non-physician health professionals has rapidly increased in the last decade, not only in Maputo but also in the northern and central parts of the country [9]. While a previous multi-country study, which included Mozambique, examined the job preferences of female nurses working in maternal and child health (enfermeiras de saúde materno-infantil) [10], the job preferences of graduate-level non-physician health professionals are yet to be examined in detail.

The study used a discrete choice experiment (DCE) to elicit the preferences of non-physician health professionals. DCEs are a quantitative method for eliciting preferences. The technique assumes that individual decisions about a good or service are determined by the attributes or characteristics of that good or service [11]. Respondents are asked to choose between hypothetical scenarios that are described by several attributes. The method is increasingly used in health economics as a means of considering the views of individuals when making decisions on health care planning, policy and resource allocation [12]. The use of DCE in health sector research in low- and middle-income countries remains recent and limited to certain areas, such as the motivation and retention of medical doctors and nurses [13-15]; the relative importance of criteria for priority setting by policy makers, health administrators or those involved with HIV/AIDS interventions [16-18]; and people’s preferences for types of health care services and health care service quality [19-21].

There has been a rapid increase in the number of studies using DCEs to investigate health workforce policy in LMIC settings. Recent literature reviews on the use of DCE in human resources for health indicate that (1) doctors or medical students were the most studied cadre; (2) while salary improvement can enhance the motivation and retention of health professionals in rural and remote areas, the provision of career development opportunities and professional and personal support can be equally important to address the issue of health professionals shortages in rural and remote areas; and (3) the relative importance of different types of non-pecuniary incentives vary according to context, type of health professional and study designs and objectives [13-15]. Knowledge gaps exist on the combination of financial and non-financial incentives that works most effectively for what group of people and in what context.

## Methods

### Study design

A DCE was undertaken to examine the relative importance of eight work-related attributes: place of work, monthly salary, provision of housing, access to a loan for the purchase of a house or land, access to formal education providing opportunities for promotion, opportunities for skills development, availability of equipment and medicine in health facilities and the opportunity for private practice. The study also determined the following: (1) the trade-offs between attributes (e.g. how much salary a respondent would be willing to sacrifice in order to gain improvements in other aspects of their job) and (2) the probability of job take-up as attribute levels change.

As qualitative methods have recently been recognized as a useful tool for defining attributes and levels, developing questionnaires and improving the validity of DCE studies [12,22], to assist in the development of relevant attributes and levels, at the commencement of the study, a number of individual interviews were undertaken with health workers to explore issues associated with working as a public sector health professional (including working conditions, incentives, job location and career development). This DCE study is part of an overarching project that will assist the Ministry of Health to develop a retention strategy for non-physician health professionals. In the first phase of the project, 122 public sector health professionals undertook a survey on their work conditions. The survey used both closed- and open-ended questions to explore what respondents valued in their work environments, and the survey results helped in identifying the key themes from which the DCE attributes and levels were established. In addition, an extensive review of published and grey literature, such as empirical studies, policy documents and government reports (both in English and Portuguese), was undertaken to determine the attributes and levels to be used in the study. Specifically, the study considered the attributes included in the recent multi-country study on the job preferences of mid-level health workers in Malawi, Mozambique and Tanzania [10] and the policy document reporting on group interviews pertaining to health workforce retention policies. Finally, several discussions with the Ministry of Health officials responsible for health workforce policy in Mozambique were held to determine the relevance of the potential attributes to the country’s health system reform policy. The selection and definition of the attributes reflected the government-level discussion on options for the health workforce retention policy. Independence and inter-relationships among the attributes was also considered. The final set of attributes and attribute levels is presented in Table 1.

**Table 1:** **Attributes and attribute levels**

**Attribute**	**Levels**	**Description**
Place of work	Rural	District capital
	Urban II	Provincial capital
	Urban I	Maputo City
Monthly salary	MZM 20 000	Base calculated as the mean official minimum monthly salary rates for N1 and N2 health professionals
	MZM 30 000	Base plus 50%
	MZM 40 000	Base plus 100%
Housing	No housing	No house is provided—housing is considered as a worker’s responsibility to be paid for at a worker’s own cost
	Government housing	Basic government housing is provided
Access to a loan for the purchase of a house or land	No access to a housing/land loan	No support provided to access a loan for the purchase of a house or land
	Access to housing/land loan provided	Access to a loan for the purchase of a house or land is provided
Formal education providing opportunities for promotion	No formal education offered	No opportunities for formal education are offered
	Formal education offered after 5 years of work	Opportunities for formal education, which may lead to a promotion to a higher level within the same occupational area, are offered after 5 years of work
Skills development	No in-service training	No in-service training offered
	Regular in-service training	On-going, short-term training courses and regular supportive supervision are provided
Availability of equipment and medicine	Inadequate	Medical equipment, drugs and facility standards do not allow the provision of a basic package of health services
	Adequate	Medical equipment, drugs and facility standards allow the provision of a basic package of health services
Private practice	Private practice is only allowed after the official work hours at public facility	Public sector health workers are only allowed to work in the private sector after the official work hours at public health facilities.
	Part-time private practice is allowed	Public sector heath workers are allowed to practice in the private sector on a part-time basis (maximum 20 h per week).

Hypothetical alternatives were generated from the attributes and levels and combined to create choice sets. The study used a ‘forced’ choice approach (i.e. respondents were asked to choose between two job profiles: job A or job B). While the inappropriate use of forced choices may result in biases in parameter estimates (e.g. individuals may be forced to select a job when, in reality, they would choose not to take either of the offered positions), there are a number of potential drawbacks of using opt-out choices in a DCE, particularly as respondents may select the opt-out option not because it provides the highest utility among the alternatives but to avoid having to make a difficult decision about the available options. In addition, allowing respondents to opt-out of making a choice provides less information on respondents’ relative preferences for the attributes in the hypothetical alternatives [15]. A fractional factorial design was used to consider a manageable selection of the possible alternatives. The study used a design catalogue reproduced from Kocur et al. [23], utilizing a ‘fold over’ design to create 16 choice sets. In addition, two warm-up choices were included in the questionnaire to familiarize respondents with the question design. Both orthogonality (attributes are statistically independent of one another) and level balance (attributes appear an equal number of times) in the questionnaire were ensured. The choice sets form the basis of the DCE questionnaire. The questionnaire also included a number of questions on respondent biographical information to enable the analysis of the impact of individual characteristics on the choices made. The questionnaire was prepared in English and translated into Portuguese. The translated questionnaire was back-translated to validate the translation quality. After four interviewers undertook a 2-day interview-training programme, the questionnaire was pilot-tested on 30 respondents in Maputo City.

### Data collection and analysis

Data collection took place in four Mozambique provinces: Maputo City, Maputo Province, Sofala and Nampula. DCE questionnaires were administered to all N1 and N2 health professionals and ‘mid-level specialists’ (i.e. non-physician health professionals who have undertaken specialized training; the N1 category indicates a full university degree with a minimum of 5 years of study; the N2 category indicates a minimum of 3 years of study; the ‘mid-level specialist’ category indicates a minimum of 1 year of specialized training) working in central, provincial and district hospitals and in the provincial directorate at the time of the study (*N* = 334). The Ministry of Health, Instituto de Ciências de Saúde de Maputo (ICSM) and health professional associations were consulted about the recruitment of the study participants. In Maputo City, the city health directorate assisted in the development of a list of N1 and N2 health professionals and mid-level specialists. In the provinces outside Maputo City, the provincial health directorate provided a list of N1 and N2 health professionals and mid-level specialists for use by the study team. The N1 and N2 health professionals and mid-level specialists sampled in the study included nurses, midwives, laboratory technicians, pharmacists, psychologists and hospital administrators. In addition, questionnaires were administered to 123 N1 and N2 students so that the study could compare the results of those who have work experience with those who do not in order to determine how new N1 and N2 graduates can be attracted to work in rural posts. The Ministry of Health identified facilities that train N1 and N2 health professionals. These facilities then assisted in the compilation of a list of N1/N2 students. The field data collection team interviewed all available N1/N2 students from the list who were in the last 2 years of their study.

A team of four people, made up of one coordinator and three interviewers, was formed to undertake the questionnaire survey. The survey team received an interview-training session that involved a general overview of the study, a detailed review of the questionnaire, instruction on questionnaire administration and role-playing of the questionnaire. After the data collection team established a list of possible study participants, the team visited the workplace or training location indicated on the list. Self-administered questionnaires were undertaken at the work place/training location of the respondents. The interviewers closely attended groups averaging in 10 respondents (minimum 2 and maximum 20, determined by the number of respondents in a work/training place) and comprehensively explained the choice tasks and other questions in the questionnaire. There are some cases where an individual self-administered questionnaire occurred (attended by an interviewer).

On completion of the survey of both workers and students, data were independently double-entered into files by two locally trained data capturers and checked for consistency using EpiData. The data were transferred to STATA for analysis.

Drawing on Lancaster’s economic theory of value [24], DCEs assume that individuals derive utility from the attributes of the commodity being valued and that individuals’ preferences are revealed through their choices. Analysis of DCE data utilizes Random Utility Theory [25,26] which states that while an individual knows the nature of their utility function, it cannot be observed by researchers. Consequently, the utility function is modelled as a systematic (explainable) component and a random (unexplainable) component. In this research, the systematic component, which can be estimated, was used to provide quantifiable information on the relative importance of job attributes. Given the random component, DCE response data was analysed within a probabilistic framework and the model used to predict how choices change in response to attribute changes. The random component of the utility function was assumed to follow a logistic distribution. A conditional logit model was used to analyse the choice response data. All variables, other than the salary attribute which was treated as continuous variable, were dummy coded. Both the sub-group analysis (of respondents’ personal characteristics) and the entire population analysis were undertaken on the data gathered from the four provinces. It has been argued that heterogeneity in attribute weights can be accounted for by a scale effect across individuals, which may invalidate comparison of sub-groups [27,28].

Willingness to pay (WTP) attaches a monetary value to marginal improvements in job attributes. To estimate the trade-offs that respondents are willing to make between attributes, WTP for marginal improvements in the attributes and associated confidence intervals were estimated for all attributes. WTP estimates were calculated as the ratio of the coefficient of interest to the negative of the coefficient on the salary attribute [29].

In addition, the probability of making a particular job choice was calculated to predict uptake rates for defined rural jobs, particularly those with improved key job attributes.

To better understand the quantitative results of the analysis, discussions were held with the data collection team to determine how the key findings from the analysis related to the context where the DCE was undertaken, based on their engagement with respondents during the data collection.

The study received ethical and research approvals from the Ministry of Health in Mozambique (ComitéNacional de Bioética para Saúde).

## Results

### Characteristics of respondents

The survey included a total of 490 respondents: 334 non-physician health professionals with university training, 123 students who were being trained to become N1 and N2 health professionals and 33 did not specify (Table 2). The sample comprised 47% male respondents and 52% female respondents. Of the respondents, 34% were 20–29 year olds, 33% were 30–39 year olds and 32% were 40 years old and above. The birthplace of the sample population was almost equally distributed with 28% born in Maputo City, 33% in a provincial capital and 33% in a district. Of the students, 32% were studying in Maputo City and 60% in other places.

**Table 2:** **Respondent characteristics**

**Variables**	**Frequency**	**%**
*N*	490	
Sex		
Male	229	47%
Female	256	52%
No response	5	1%
Birth place		
Maputo City	135	28%
Provincial capital	161	33%
District	160	33%
No response	34	7%
Age group		
20–29 years old	169	34%
30–39 years old	163	33%
40–49 years old	115	23%
50 years old and above	43	9%
Current status		
Health professional	334	68%
Student	123	25%
No response	33	7%

### Entire population analysis

In the entire population analysis (Table 3), all attributes were statistically significant, indicating that they impact on the probability of choosing a job. All ‘improved’ levels of the attributes had positive coefficients, implying that they positively impact on the decision to choose a job. These results support the theoretical validity of the model. The provision of basic government housing had the greatest impact on the decision to choose a job for N1 and N2 health professionals and mid-level specialists. This result was consistent across the entire population analysis and in the sub-group analysis of health professionals and students. Across the entire study, the coefficient for working outside of Maputo (working either in a district or provincial capital), were both significant and negative, indicating that respondents prefer not to work in these places.

**Table 3:** **Results from conditional logit model**

	**Variables**	**Model 1**	
		**Coefficients**	**SE**
Salary	Salary	0.00007***	0.00000
Place of work: district capital	Work district	−0.33563***	0.04959
Place of work: provincial capital	Work province	−0.10953**	0.03397
Housing: basic government housing is provided	Housing provided	0.54174***	0.03116
Access to a loan for the purchase of a house or land: access to a loan for the purchase of a house or land is provided	Loan provided	0.31735***	0.02690
Formal education providing opportunities for promotion: opportunities for formal education are offered after 5 years of work	Education provided	0.51144***	0.03448
Skills development: on-going, short-term training courses and regular supportive supervision are provided	Training provided	0.27148***	0.02515
Availability of equipment and medicine: medical equipment, drugs and facility standards allow the provision of a basic package of health services	Medicine/equipment available	0.33995***	0.03020
Private practice: public sector heath workers are allowed to practice in the private sector on part-time basis (maximum 20 h per week)	Private practice allowed	0.08811***	0.02211
Constant		−0.01912	0.02746
Number of groups		488	
Number of observations		15 458	
Log likelihood		−4 597.9504	
Wald chi-square		914.98	
Prob > chi-square		0.0000	

***P < 0.001, **P < 0.01.

### Sub-group analysis

Tables 4, 5 and 6 provide information on the interaction of all health workforce retention policy option coefficients with dummy variables for health professionals and students, the age group of health professionals and place of birth.

**Table 4:** **Results from conditional logit model, including interactions with the health professionals and students**

	**Model 2**		
	**Coefficient**	**SE**	***P*** **> |** ***z*** **|**
Salary	0.00006	9.79e − 06	0.000
Salary*health professionals	0.00001	0.00001	0.217
Place of work: district capital	−0.01274	0.08298	0.878
Place of work: district capital*health professionals	−0.46284	0.10504	0.000
Place of work: provincial capital	0.10538	0.06535	0.107
Place of work: provincial capital*health professionals	−0.30624	0.07642	0.000
Housing	0.53866	0.06060	0.000
Housing*health professionals	0.00126	0.07085	0.986
Access to a loan	0.38210	0.05889	0.000
Access to a loan*health professionals	−0.07671	0.06722	0.254
Formal education	0.51927	0.06637	0.000
Formal education*health professionals	−0.00102	0.07890	0.990
Skills development	0.26677	0.04949	0.000
Skills development*health professionals	0.01989	0.05856	0.734
Availability of equipment and medicine	0.25184	0.05697	0.000
Availability of equipment and medicine*health professionals	0.12082	0.06803	0.076
Private practice	0.10305	0.04295	0.016
Private practice*health professionals	−0.02353	0.05097	0.644
Const	−0.02475	0.02854	0.386
Number of groups	455		
Number of observations	14 408		
Log likelihood	−4 269.1452		
Wald chi-square	919.02		
Prob > chi-square	0.0000

**Table 5:** **Results from conditional logit model, including interactions with the age group of health professionals**

	**Model 3**		
	**Coefficient**	**SE**	***P*** **> |** ***z*** **|**
Salary	0.00007	0.00001	0.000
Salary*30–39 years old	−9.45e − 06	0.00002	0.535
Salary*40 and above	2.67e − 06	0.00002	0.861
Place of work: district capital	−0.47022	0.11351	0.000
Place of work: district capital*30–39 years old	−0.11078	0.16615	0.505
Place of work: district capital*40 and above	0.08731	0.14913	0.558
Place of work: provincial capital	−0.27954	0.08120	0.001
Place of work: provincial capital*30–39 years old	0.05551	0.10771	0.606
Place of work: provincial capital*40 and above	0.17654	0.10206	0.084
Housing	0.70908	0.07968	0.000
Housing*30–39 years old	−0.13419	0.10567	0.204
Housing*40 and above	−0.27447	0.09409	0.004
Access to a loan	0.28249	0.06594	0.000
Access to a loan*30–39 years old	0.05479	0.08769	0.532
Access to a loan*40 and above	0.01605	0.08234	0.845
Formal education	0.63018	0.09146	0.000
Formal education*30–39 years old	−0.14652	0.11634	0.208
Formal education*40 and above	−0.14095	0.11224	0.209
Skills development	0.36585	0.06287	0.000
Skills development*30–39 years old	−0.14554	0.08031	0.070
Skills development*40 and above	−0.07032	0.08063	0.383
Availability of equipment and medicine	0.43079	0.06908	0.000
Availability of equipment and medicine*30–39 years old	−0.04824	0.09643	0.617
Availability of equipment and medicine*40 and above	−0.09740	0.09032	0.281
Private practice	0.16709	0.05450	0.002
Private practice*30–39 years old	−0.11910	0.07083	0.093
Private practice*40 and above	−0.10244	0.07039	0.146
Constant	0.01077	0.03282	0.743
Number of groups	332		
Number of observations	10 484		
Log likelihood	−3 076.0471		
Wald chi-square	749.65		
Prob > chi-square	0.0000

**Table 6:** **Results from conditional logit model, including interactions with the place of birth**

	**Model 4**		
	**Coefficient**	**SE**	***P*** **> |** ***z*** **|**
Salary	0.00009	0.00001	0.000
Salary*provincial capital	−0.00003	0.00001	0.015
Salary*districts	−0.00002	0.00001	0.119
Place of work: district capital	−0.57966	0.09777	0.000
Place of work: district capital*provincial capital	0.26907	0.12477	0.031
Place of work: district capital*districts	0.40716	0.13431	0.002
Place of work: provincial capital	−0.35652	0.06233	0.000
Place of work: provincial capital*provincial capital	0.34973	0.08421	0.000
Place of work: provincial capital*districts	0.33625	0.08590	0.000
Housing	0.52938	0.06257	0.000
Housing*provincial capital	0.09460	0.08320	0.255
Housing*districts	−0.03725	0.08239	0.651
Access to a loan	0.27705	0.05452	0.000
Access to a loan*provincial capital	0.09491	0.07297	0.193
Access to a loan*districts	0.00614	0.07035	0.930
Formal education	0.47774	0.06767	0.000
Formal education*provincial capital	0.03648	0.08771	0.677
Formal education*districts	0.09914	0.09319	0.287
Skills development	0.34018	0.04542	0.000
Skills development*provincial capital	−0.07898	0.06449	0.221
Skills development*districts	−0.09011	0.06360	0.157
Availability of equipment and medicine	0.31708	0.05508	0.000
Availability of equipment and medicine*provincial capital	0.06366	0.07990	0.426
Availability of equipment and medicine*districts	−0.00663	0.07391	0.929
Private practice	0.10655	0.03819	0.005
Private practice*provincial capital	−0.02214	0.05701	0.698
Private practice*districts	−0.02237	0.05371	0.677
Constant	−0.01245	0.02846	0.662
Number of groups	455		
Number of observations	14 428		
Log likelihood	−4 260.1823		
Wald chi-square	926.15		
Prob > chi-square	0.0000		

#### Non-physician health professionals and students

Table 4 shows the results for non-physician health professionals and students, using students as the excluded group. Interactions are negative for working in districts and provinces, indicating that non-physician health professionals are less likely to want to work in rural/remote areas than students. Among the non-physician health professionals, the interactions are positive for the availability of equipment and medicine at health facilities, suggesting that this group value an adequately equipped work environment more than students do. The difference between the subgroups may be influenced by the amount of experience working at public sector health facilities where a lack of equipment and medicinal stocks produces a challenging work environment for health professionals.

#### Age group of health professionals

Variations exist between age groups in the preferences for job attributes (Table 5). The excluded group is the 20–29-year-old age group. The interaction terms are negative for the provision of basic housing among those aged 40 and above, whereas the interaction terms are negative for the provision of skills development among those 30–39 years old. The results suggest that those aged 40 and above value the provision of basic housing less than those aged 20–29 years old and those aged 30–39 value skills development opportunities less than those aged 20–29 years old. The disparity in preferences held by different age groups suggests that incentive packages should vary according to the stage of life of health professionals.

### Place of birth

Table 6 shows the results according to birthplace. The excluded group is those born in Maputo City. For those born in provincial capitals or districts, the interaction terms are positive for working in a provincial capital and/or a district capital, indicating that this group is more willing to work in rural areas than those born in Maputo City. The interaction terms are positive for the provision of formal education for those born in district locations, indicating that there is a stronger preference for formal education opportunities for this group than for those born in Maputo City.

### Willingness to pay

In the entire population analysis, a marginal improvement in non-financial incentive attributes increases the WTP as follows (Table 7):

1. Provision of basic government housing increases WTP by MZM 7 893 (95% CI 6 498, 9 287)
2. Provision of formal education increases WTP by MZM 7 451 (95% CI 6 029, 8 874)
3. Availability of equipment and medicine at a health facility increases WTP by MZM 4 953 (95% CI 3 829, 6 076)
4. Access to a loan for the purchase of a house or land increases WTP by MZM 4 623 (95% CI 3 643, 5 604)
5. Provision of skills development increases WTP by MZM 3 955 (95% CI 3 071, 4 840)
6. Permission to undertake private practice increases WTP by MZM 1 284 (95% CI 636, 1 931)

**Table 7:** **Marginal willingness to pay estimates—entire population, age group and place of birth**

	**All**	**Age group**			**Place of birth**		
	**(** ***N*** **= 490)**	**20–29 years old (** ***N*** **= 169)**	**30–39 years old (** ***N*** **= 163)**	**40 and above (** ***N*** **= 158)**	**Maputo City (** ***N*** **= 135)**	**Provincial capital (** ***N*** **= 161)**	**Districts (** ***N*** **= 160)**
Place of work: district capital	−4 890 (−6 382, −3 398)	−6 521 (−9 755, −3 286)	−9 213 (−13 684, −4 743)	−5 022 (−7 804, −2 240)	−6 683 (−9 119, −4 247)	−5 755 (−8 797, −2 712)	−2 700 (−5 525, 124)
Place of work: provincial capital	−1 596 (−2 579, −612)	−4 273 (−6 648, −1 897)	−3 560 (−5 939, −1 181)	−1 101 (−2 826, 623)	−3 675 (−5 325, −2 025)	749 (−2 839, 1 340)	−439 (−2 216, 1 338)
Housing: basic government housing provided	7 893 (6 498, 9 287)	9 324 (6 014, 12 635)	9 109 (5 636, 12 581)	5 978 (3 884, 8 071)	6 666 (4 593, 8 740)	10 878 (7 349, 14 407)	7 494 (4 995, 9 992)
Access to a loan: access to a loan for the purchase of a house or land is provided	4 623 (3 643, 5 604)	3 694 (1 661, 5 728)	5 345 (2 902, 7 788)	4 061 (2 495, 5 628)	3 403 (1 847, 4 958)	6 494 (4 104, 8 884)	4 312 (2 651, 5 974)
Formal education: opportunities for formal education are offered after 5 years of work	7 451 (6 029, 8 874)	8 379 (4 595, 12 162)	7 665 (4 542, 10 788)	6 651 (4 231, 9 071)	5 849 (3 687, 8 011)	9 016 (5 953, 12 079)	8 805 (5 890, 11 720)
Skills development: on-going, short-term training courses and regular supportive supervision are provided	3 955 (3 071, 4 840)	4 882 (2 495, 7 268)	3 492 (1 780, 5 204)	4 017 (2 378, 5 657)	4 150 (2 731, 5 568)	4 580 (2 635, 6 524)	3 812 (2 194, 5 431)
Availability of equipment and medicine: medical equipment, drugs and facility standards allow the provision of a basic package of health services	4 953 (3 829, 6 076)	5 787 (3 061, 8 512)	6 063 (3 344, 8 783)	4 503 (2 510, 6 495)	3 818 (2 187, 5 449)	6 757 (4 005, 9 509)	4 753 (2 800, 6 706)
Private practice: public sector health workers are allowed to practice in the private sector on a part-time basis (maximum 20 h per week)	1 284 (636, 1 931)	2 099 (613, 3 585)	759 (−693, 2 210)	946 (−270, 2 163)	1 468 (516, 2 421)	1 374 (115, 2 863)	1 262 (90, 2 435)

Working in a district capital reduces WTP by MZM 4 890 (95% CI −6 382, −3 398), and working in provincial capital reduces WTP by MZM 1 596 (95% CI −2 579, −612).

The figures in parenthesis indicate the 95% confidence interval for the WTP estimates. As WTP is derived from the ratio of two random variables, WTP is itself a random variable. Consequently, confidence intervals were determined for WTP estimates, and the results indicate the statistical significance of the WTP estimates.

Overall WTP is calculated as the sum of the WTPs for both the positive levels and the negative levels of attributes. Individuals are more likely to accept a job if the overall WTP for a defined position is positive. For example, for a job offered in a district capital (WTP MZM −4 890), with no access to a housing loan (WTP MZM −4 623), no skills development provided (WTP MZM R −3 955) and private practice only allowed after official work hours at public facility (WTP MZM −1 284), if housing benefits are provided (WTP MZM 7 893), professional development opportunities provided (WTP MZM 7 451) and medical equipment and drugs sufficient for the provision of a basic package of health services (WTP MZM 4 953), the overall WTP for the job at the specified public facility becomes positive (WTP MZM 5 545), indicating that people would take the job (because they would ultimately be better off). The overall WTP result indicates that when the three most preferred incentives are provided (i.e. housing benefits, formal education opportunities and an adequate supply of medical equipment and drugs at a health facility), non-physicians are likely to accept a position at the specified public health facility even if the job is in a rural area and incentives other than the three most preferred are not provided.

The WTP results for those born in district locations (i.e. rural areas) indicate that if only the two most preferred incentives are provided (i.e. provision of housing benefits and professional development opportunities), other incentives not provided and the job located in a district capital, the overall WTP for the job would be positive, and the candidate would take the job at the specified public health facility.

### Uptake rates of choosing a job in rural/remote areas

The estimation of the probability of choosing a defined job confirmed that housing benefits, professional development opportunities and an adequate supply of medical equipment and drugs at a health facility are the most highly valued incentives influencing job uptake. The uptake rate for a rural job that offers the three most preferred incentives (housing benefits, professional development opportunities and an adequate supply of medical equipment and drugs at a health facility when the job is offered in a district capital with monthly salary of MZM 20 000) is predicted to be 85.6% whereas the uptake rate for a rural job that offers other incentives (a housing loan, skills development and private practice when the job is offered in a district capital with monthly salary of MZM 20 000) is estimated to be 58.6%.

Furthermore, the uptake rate of a rural job offering the three most preferred incentives is predicted to be 87.9% for those born in district locations, higher than the overall estimated uptake rate of choosing a rural job that provides the three most preferred incentives.

## Discussion

The results of this study help identify priorities for the human resources for health (HRH) reforms currently on the policy agenda in Mozambique and add evidence to the literature on the job preferences of health care providers in LMIC, examining the relative importance of factors influencing health worker choices. The marginal change in the provision of basic government housing has the greatest impact on the probability of choosing a job at a public health facility, followed by the provision of formal education opportunities and the availability of equipment and medicine at the health facility. If these forms of incentives are provided, non-physician health professionals would take up the job at the specified public health facility even if it is in a rural area.

The results support current evidence by indicating that (1) non-pecuniary incentives are significant determinants in choice of job; (2) the combination of financial and non-financial incentives needs to be considered in order to retain health workforce in a rural, remote area; and (3) it is important to investigate the preferences of health worker sub-groups [11,14,23]. However, our study results slightly contradict a previous multi-country study, which included Mozambique, that examined job preferences for nurses and midwives and found that the provision of housing has limited impact on job choices [10].

This study was undertaken within an overarching project that aimed to develop retention strategies for non-physician health professionals. The results of this DCE study, qualitative studies and a policy document review were all used by the study team to produce recommendations for a range of incentive packages and strategies focusing on the provision of housing benefits (including the provision of basic government housing and access to a housing loan) and professional development opportunities (including the provision of formal education, which can lead to career and skills development) with specific incentives for non-physician health professionals assigned to rural/remote areas. The project team also undertook an estimation of the resources required to implement the suggested strategies and found that the affordability of the strategies falls within the limits of the national HRH plan (2008–2015).

In recent times, the research community has been debating (1) the extent to which DCE can be used to advise policy-makers, given that DCEs are complex tools to design for respondents to complete and for researchers to analyse and (2) the information that DCEs can provide to policy makers and how DCE compares with alternative approaches to assist in the decision-making process [4]. Our study offers a positive example where the DCE results provided useful information to assist policy decision making, and together with the other types of research, the DCE results were used in the development of the human resource strategy.

When designing the DCE, the study team engaged in significant discussion before deciding the housing attributes for inclusion in the DCE. The team worked closely with the Ministry of Health who requested that two housing-related attributes be included in the DCE (i.e. provision of government housing and access to loan for the purchase of a house or land). The Ministry regarded both policy options to be important for the development of recruitment and retention policies for rural health professionals. The government housing attribute was described by the Ministry as ‘the provision of basic housing by government for the period of the job assignment’, contrasting to the position that housing is paid for by the worker. Access to a housing loan differs to the provision of housing attribute in that the government provides access to a loan for the purchase of a house or land. Given that 54.7% of the population in Mozambique lives under the poverty line, access to a loan/credit to purchase property is considered a valuable opportunity, and this value extends beyond the period of the appointment to a particular post. It is possible for both attributes to be provided as part of one job post—a particular job can provide basic government housing but can also provide access to a loan/opportunities to purchase a house in the place the incumbent is assigned, or elsewhere, or to buy a house that can better accommodate the incumbent’s family.

As the results from the sub-group analysis suggest that job preferences vary according to stage of life (i.e. between age groups and level of experience as a health professional), incentive packages should also vary according to the health professional’s life stage. The results indicate that an individual’s work experience influences his/her preferences [14]. While salaries for health workers often vary according to level of education, the results of the sub-group analysis suggest that, in order to encourage health professionals to work in rural, remote areas, the incentive package (a combination of both financial and non-financial incentives) may need to be tailored to meet the personal and professional needs of those allocated to rural and remote positions and should vary according to the stage of life of the professionals being targeted.

In addition, as the sub-group analysis suggests that those born in provincial capitals or districts are more willing to work in rural areas than those born in Maputo City, strategies for recruiting non-clinical professionals to work in rural/remote areas should also consider birthplace. A global task force established by the World Health Organization (WHO) in 2010 to examine adverse effects of intra-country relocation of health workers released 16 evidence-based recommendations for improved retention of health workers in remote and rural areas [2,3], one of which was to target admission policies to enrol students with a rural background in education programmes for health disciplines. Our finding supports the direction that the international community is recommending to address the long-standing problem of encouraging health workers to work in rural and underserved areas.

For the sub-group analysis, interaction terms were estimated to determine how the effect of one independent variable on the dependent variable depends on the magnitude of another independent variable. Recent literature on DCE application in the health sector notes that only a small number of health economics DCEs include interaction terms in the analysis [12,14]. While the use of interaction terms in health economics DCEs is an area for further exploration, preferences for attributes by respondents may depend on the level of other attributes and the inclusion of selected interactions adds value to DCE studies. On these grounds, the authors included interaction terms in the study. Separate analysis by sub-groups was undertaken (separately estimating the main effects of attributes according to sub-group using conditional logit model and conducting Wald tests for true differences) and similar results obtained.

Although statistically significant, the relative importance of the salary attribute in our study is consistently low across all models estimated. DCE studies on human resources for health indicate that non-monetary job attributes are significant determinants of individuals’ job choice and can be more influential than monetary incentives [13,30]. Our study confirms the importance of non-monetary job attributes and reveals the need to develop incentive packages with a mix of monetary and non-monetary incentives. Further research is required to determine the most effective mix of incentives to attract the target health professionals in the context in which the incentive package operates.

In addition, our results show that the private practice attribute is less important than the other non-monetary attributes when non-clinical health professionals choose rural employment. The results may have been influenced by the context of rural, underserved areas in Mozambique, where there is less demand for private practice due to the limited economic resources of and stronger preferences for traditional medicine among people living in those areas. Engagement in private practice is linked to structure and features of local markets for health services [31]. How this affects the development of human resource recruiting policies requires further exploration.

There are a number of methodological limitations in this study. Firstly, small sample sizes for some of the sub-groups may have limited the statistical power of the analysis. Secondly, the DCE questionnaire suffered inconsistent administration techniques, and while most of the DCE questionnaires were administered with close attention from interviewers, there were a number of self-administered questionnaires that were undertaken without an interviewer present, which may have affected the level of comprehension between groups when answering the choice set questions in the questionnaire. Thirdly, while heterogeneity of the target group (i.e. N1 and N2 and mid-level specialist categories of health professionals) should have been examined using sub-group analysis, small and skewed sample sizes for the types of the health professions did not allow further statistical analysis on the health professional sub-groups. Lastly, in the WTP estimation, the salary attribute was entered as a continuous variable and a marginal rate of substitution was calculated, dividing the marginal utility of an attribute over the marginal utility of the salary attribute [15,32]. However, respondents made choices on the levels of the salary attributes, and the non-continuous nature of the salary attribute may have limited the analysis of the WTP results.

Lastly, integration of qualitative approaches throughout the study process, including in the establishment of attributes and attribute levels and generation of the questionnaire and consultation processes to obtain a deeper understanding of the study results, is increasingly recognized as important in DCE studies [12,33]. Consequently, the process of undertaking DCE is considered to be a mixed method approach [34,35]. In this study, a qualitative approach was used to establish the attributes and levels and to develop deeper understanding of the quantitative results in the context in Mozambique. While DCE provides a unique opportunity to use both qualitative and quantitative approaches together in the study process, further research is required to determine how the mixed method approach can be best used to tailor DCE to particular study settings.

## Conclusion

The results from the entire population analysis indicate that the provision of basic government housing has the greatest impact on the probability of choosing a job at a public health facility, followed by the provision of formal education opportunities and the availability of equipment and medicine at the health facility. If these incentives are provided, non-physician health professionals are likely to take specified jobs at public health facilities, even though the work may be located in a rural area. Additionally, the sub-group analysis suggests that job preferences vary according to stage of life and that incentive packages should consider the life stage of health professionals. Recruitment strategies for non-clinical professionals to work in rural/remote areas should also consider birthplace as those who were born in rural/remote area are more willing to work in rural/remote locations. Despite some methodological limitations, the results of this DCE study, conducted in a short period and at a relatively low cost, have been used to formulate the incentive packages and strategies to be used when assigning non-clinician health professionals to rural/remote areas of Mozambique.
